# Evaluation of alternative transfusion triggers in hemodynamically stable, non-ventilated cancer patients: a prospective observational study

**DOI:** 10.1038/s41598-025-32630-6

**Published:** 2025-12-19

**Authors:** Ardak Arynov, Johannes Gratz, Barbara Kabon, Dilyara Kaidarova, Alima Satanova, Evgeni Brotfain

**Affiliations:** 1 Kazakh National Medical University named after S.D. Asfendiyarov, Tole bi str. 88, Almaty, Kazakhstan; 2 Department of Anesthesiology and Intensive Care Medicine, Charité – Universitätsmedizin Berlin, Campus Benjamin Franklin, Berlin, Germany; 3 Department of Anaesthesia, Intensive Care Medicine and Pain Medicine, Clinical Division of General Anaesthesia and Intensive Care Medicine, Medical University of Vienna, Spitalgasse 23, 1090 Vienna, Austria; 4 Department of Gynecologic Oncology, Kazakh Institute of Oncology and Radiology, Abay av. 91, Almaty, Kazakhstan; 5 Division of Anesthesia and Critical Care, Soroka University Medical Center, Ben Gurion University of the Negev, Yitzhack I. Rager Blvd 151, Be’er Sheva, Israel

**Keywords:** Anemia, Blood transfusion, Transfusion triggers, Oxygen extraction, Cancer, Health care, Medical research, Oncology

## Abstract

Anemia is highly prevalent among oncological patients and is often managed with red blood cell transfusions. Current guidelines predominantly rely on hemoglobin levels to guide transfusion, but hemoglobin alone may not accurately reflect oxygen delivery. Physiological triggers, particularly the oxygen extraction ratio (O_2_ER), could provide a more individualized transfusion strategy. This prospective, single-center, observational study included 107 clinically stable adult oncology patients at the Kazakh Institute of Oncology and Radiology. All patients required red blood cell transfusion based on a restrictive trigger (Hb 70 g/L). Patients were stratified into two groups according to their baseline O_2_ER (≤ 35.4% or > 35.4%). The primary outcome was the change in O_2_ER before and one hour after transfusion. We also measured changes in central venous oxygen saturation (ScvO_2_), central venous oxygen partial pressure (PvO_2_), arteriovenous oxygen difference (A-V O_2_diff), lactate, and veno-arterial carbon dioxide difference (ΔCO_2_). Patients with higher baseline oxygen extraction ratio (O_2_ER > 35.4%) demonstrated more pronounced improvements in oxygenation parameters—including O_2_ER, ScvO_2_, PvO_2_, A‑V O_2_ diff, and ΔCO_2_—following transfusion, compared to patients with O_2_ER ≤ 35.4%. Although baseline hemoglobin levels and post-transfusion increases in hemoglobin were similar between groups, significant differences were observed across all physiological markers of oxygen transport. Correlation analyses showed significant associations between baseline O_2_ER and changes in PvO_2_, ScvO_2_, A‑V O_2_ diff, ΔCO_2_, and lactate. In contrast, baseline hemoglobin demonstrated no significant correlations with most physiological response parameters, except for a moderate but statistically significant correlation with the change in blood lactate levels. Lactate levels remained within normal limits in the majority of patients, indicating that critical oxygen delivery thresholds were not reached. In stable cancer patients with anemia, hemoglobin levels alone may not adequately capture oxygen delivery status. The oxygen extraction ratio and other physiological triggers offer valuable insights into which patients may benefit most from transfusion. Our findings support a more individualized transfusion strategy, though larger randomized controlled trials are warranted to confirm the safety and efficacy of physiological triggers in broader patient populations.

*Trial Registration* The study protocol was retrospectively registered on ClinicalTrials.gov (NCT06952361, Initial Release 04/23/2025).

**Supplementary Information:**

The online version contains supplementary material available at 10.1038/s41598-025-32630-6.

## Background

The prevalence of anemia among patients varies depending on their underlying disease and other contributing factors. In the perioperative period and in critically ill patients, especially among cancer patients, anemia is common, with reported prevalence reaching up to 90%, and has also been associated with adverse clinical outcomes^1–7^. The transfusion of packed red blood cells remains the primary treatment of anemia. However, also transfusion itself has been associated with poorer short—and long-term outcomes in the perioperative period, particularly in the context of aortic-, major abdominal and cardiac surgery^8–10^. Thus, current transfusion guidelines from leading professional societies, consistently endorse a restrictive transfusion strategy for most hemodynamically stable and non-bleeding patients, generally using a hemoglobin threshold of 70–80 g/L.^11–15^. While this threshold might be higher in patients with cardiovascular disease or undergoing cardiac or orthopedic surgery, specifically in oncological patients, a restrictive transfusion approach might be beneficial, as reducing the number of transfusions may help decrease transfusion-related complications, mitigate the negative impact on overall mortality, and potentially lower the risk of recurrence in certain types of cancer^16–18^.

While the indication for blood transfusion in most current guidelines is based on absolute hemoglobin values, the European Society of Anaesthesiology and Intensive Care (ESAIC) highlights the role of physiological transfusion triggers such as central venous oxygen saturation or arteriovenous oxygen difference and all recommendations emphasize the importance of an individualized approach when making decisions about blood transfusions^11–15^. Hemoglobin levels may not adequately reflect the sufficiency of oxygen delivery and consumption. Moreover, the physiological ability to compensate for anemia through increased cardiac output may be limited in some patients, such as those with heart failure or advanced age^19–21^. Even in high-risk populations, including those in which a liberal strategy has shown benefit and others in which a restrictive approach appears favorable, evidence regarding an optimal transfusion strategy based solely on absolute hemoglobin values remains inconclusive^19,20,22–25^. Thus, the use of physiological transfusion triggers to identify individuals who benefit most from transfusion might be preferable, especially in oncological patients^26^.

In the present study, we prospectively analyzed the changes in various physiological triggers in response to blood transfusion in a cohort of hemodynamically stable cancer patients with an indication for red blood cell transfusion according to our local algorithm. The aim of this study was to prospectively assess the changes in physiological transfusion triggers in response to red blood cell transfusion in cancer patients dependent on their baseline O_2_ER.

## Patients and methods

This single-center, prospective, observational study was approved by the local ethics committee of the Kazakh Institute of Oncology and Radiology (protocol No. 2, February 4, 2019). All methods were performed in accordance with the relevant guidelines and regulations, including the Declaration of Helsinki (2013 revision). It was designed and reported in accordance with the STROBE (Strengthening the Reporting of Observational Studies in Epidemiology) guidelines.

The study was performed at the Kazakh Institute of Oncology and Radiology, Almaty city, Kazakhstan from February 2019 to December 2021. The Kazakh Institute of Oncology and Radiology is one of the main cancer centers in Kazakhstan, with a capacity of 450 beds, providing all types of oncological care to patients with malignant tumors of various localizations. A cohort of 107 anemic cancer patients who were identified as requiring blood transfusion were consecutively enrolled after meeting the inclusion criteria and providing written informed consent. The study population included cancer patients in different clinical settings, such as preoperative, postoperative, and those receiving chemotherapy. Clinical information was retrieved from our digital Medical Information System “Damu Med” (“DAMUMED” LLP, Astana, Kazakhstan).

*Inclusion criteria*

- Adult cancer patients (≥ 18 years)
- Hemodynamically stable
- Not requiring any respiratory support
- Presence of a central venous catheter in the superior vena cava

*Exclusion criteria*

- Hemodynamic instability requiring inotropic or vasopressor support
- Acute bleeding or any form of shock
- Refusal to participate
- Hematological malignancies
- Pediatric patients (< 18 years)
- Pregnancy

### Blood transfusion protocol

The primary indication for blood transfusion in patients included into this study was a hemoglobin level of 70 g/L. Hemoglobin level was measured as a part of standard care and not for study purposes. Hemoglobin levels were measured prior to the decision to transfuse in all included patients. The transfusion was performed in accordance with the current standards for blood transfusion in the Republic of Kazakhstan.

While most patients received one unit of red blood cells, in some cases two units were administered according to the institutional protocol, based on the baseline hemoglobin level and clinical judgment. The mean storage duration of the transfused red blood cell units was 10–12 days. Because O_2_ER values were computed subsequently for the purpose of the present analysis, physicians involved in patient care were not aware of these values.

### Measurement of physiological parameters

To evaluate the physiological impact of blood transfusion, key physiological parameters and hemodynamic variables (heart rate, mean arterial pressure) were assessed before and one hour after transfusion using arterial and central venous blood samples; blood samples were obtained via a single arterial puncture. Calculated physiological indices included the following:

Oxygen Extraction Ratio:

$${\mathrm{O}}_{2} {\mathrm{ER}} = \frac{{{\mathrm{CtaO}}_{2} - {\mathrm{CtvO}}_{2} }}{{{\mathrm{CtaO}}_{2} }} \times 100$$

Oxygen content (CtO_2_, mL O_2_/100 mL):

$${\mathrm{CtO}}_{2} = 1.34 \times {\mathrm{Hb}} \times \left( {\frac{{{\mathrm{SxO}}2}}{100}} \right) + 0.031 \times {\mathrm{PxO}}_{2}$$

Arterio-venous oxygen content difference (A-V O_2_ diff, mL O_2_/100 mL):

$${\text{A - V}} {\mathrm{O}}_{{2}} {\mathrm{diff}} = {\mathrm{CtaO}}_{{2}} - {\mathrm{CtvO}}_{{2}}$$

Veno-arterial carbon dioxide difference (ΔCO_2_, mmHg):

$$\Delta {\mathrm{CO}}_{{2}} = {\mathrm{PvCO}}_{{2}} - {\mathrm{PaCO}}_{{2}}$$

CtaO2 - arterial oxygen content (mL O_2_/100 mL), CtvO2- central venous oxygen content (mL O₂/100 mL), Hb - hemoglobin concentration (g/L), SxO2 - oxygen saturation (%), PxO2 - partial pressure of oxygen (mmHg) where: for arterial blood: SxO2=SaO2, PxO₂=PaO2, for central venous blood: SxO2 = ScvO2, PxO2 = PvO_2_, PaCO_2_ - partial pressure of carbon dioxide in arterial blood (mmHg), PvCO2 - partial pressure of carbon dioxide in central venous blood (mmHg). 

All blood gas and lactate measurements, including ScvO_2_, PvO_2_, PaCO_2_, PvCO_2_, and lactate levels, were performed using the ABL 800 FLEX analyzer (RADIOMETER, Denmark) before and 60 min after transfusion.

Patients were stratified into two groups based on the median baseline oxygen extraction ratio (O_2_ER), which was 35.4%. The Low O_2_ER group included patients with O_2_ER ≤ 35.4%, and the High O_2_ER group included those with O_2_ER > 35.4%.

### Statistical analysis

R 4.3.1 (R Foundation for Statistical Computing, Vienna, Austria) was used for statistical analysis and data visualization.

Descriptive statistics for categorical variables are presented as absolute and relative frequencies (n (%)) and for quantitative variables as median (1st–3rd quartile). The Mann–Whitney test was used for between-group quantitative variables comparison, and Fisher’s exact test was used to compare groups concerning categorical variables. Wilcoxon test was used for comparison of quantitative variables before and after the intervention. Spearman’s rank correlation coefficient (ρ) with a corresponding 95% confidence interval (95% CI) was used as a measure of monotonic association. The association was considered statistically significant at *p* < 0.05.

As this was a prospective observational study, the sample size was determined by the number of eligible patients recruited over a pre-specified period from February 2019 to December 2021. No formal sample size calculation was performed in advance, as the aim was to include all consecutive patients meeting the inclusion criteria during this period. This approach was considered sufficient for exploratory subgroup analyses.

The primary outcome parameter was the change in key physiological variables before and after transfusion, including (O_2_ER), (ScvO_2_), (PvO_2_), Lac, A-V O_2_diff, ΔCO_2_ with particular focus on their association with baseline O_2_ER levels.

## Results

### Patient selection and clinical characteristics

**Fig. 1:**
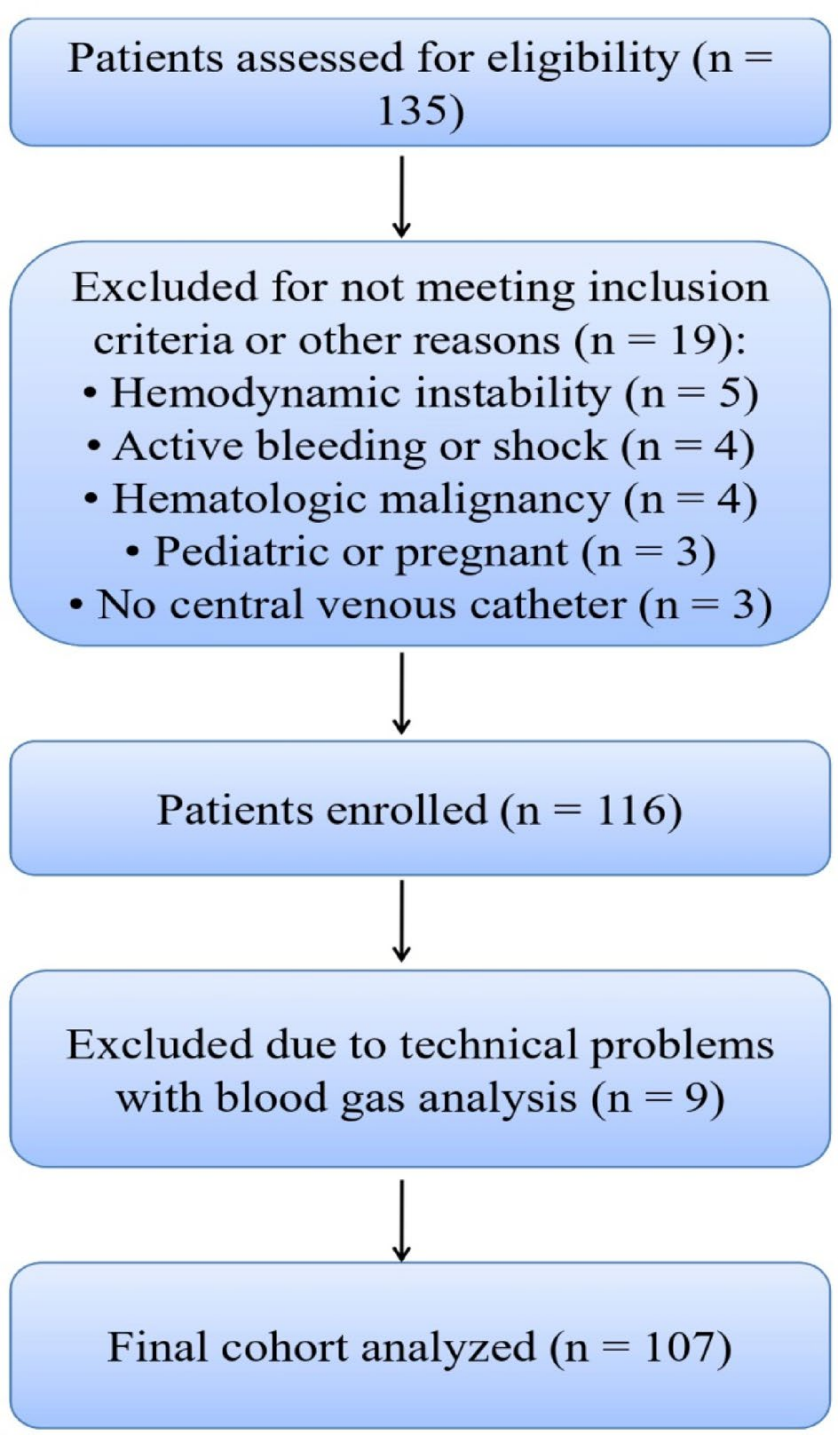
Flow chart of patient enrollment and analysis.

A total of 135 patients were assessed for eligibility between February 2019 and December 2021. Figure 1 gives an overview of patient inclusion and analyses, with the final study cohort including 107 patients. The study population consisted of adult cancer patients with anemia and a clinical indication for red blood cell transfusion. After assessing the dynamics of the studied parameters, we stratified the patients into two groups based on the median oxygen extraction ratio of 35.4% prior to transfusion. The “Low O_2_ER” group included patients with an O_2_ER ≤ 35.4%, whereas the “High O_2_ER” group included those with an O_2_ER > 35.4%. In total, 54 patients were assigned to the “Low O_2_ER” group, while 53 patients were in the “High O_2_ER” group. The demographic characteristics and relevant comorbidities of all patients, as well as those by group, are presented in Table 1. Minor or isolated diagnoses not expected to affect the course or interpretation of the study were not included.

**Table 1:** Demographic characteristics and comorbidities.

Characteristics	All patients, n = 107	“Low O_2_ER” (≤ 35.4%), n = 54	“High O_2_ER” (> 35.4%), n = 53	*p*-value
Age, (years)	57 [46‒65]	57.5 [46.3‒64]	57 [44‒66]	0.988
Sex				0.167
Women, (n, %)	66 (61.7%)	37 (68.5%)	29 (54.7%)	
Men, (n, %)	41 (38.3%)	17 (31.5%)	24 (45.3%)	
BMI, (kg/m^2^)	23.9 [21‒26.7]	24 [21.1‒26.7]	23.6 [20.7‒26.6]	0.688
*Type of cancer*				
Thoracic cancer, (n, %)	6 (5.6%)	2	4	
Gastrointestinal cancer, (n, %)	32 (29.9%)	20	12	
Genitourinary cancer, (n, %)	8 (7.5%)	1	7	
Gynecological cancer, (n, %)	22 (20.6%)	12	10	
Tumors of Bone and Soft tissue, (n, %)	30 (28%)	14	16	
Cancer of the Head and Neck, (n, %)	5 (4.7%)	3	2	
Multiple primary tumors, (n, %)	3 (2.8%)	1	2	
Breast cancer, (n, %)	1 (0.9%)	1	-	
*Co-existing disease*				
Ischemic heart disease, (n, %)	14 (13.1%)	6 (11.1%)	8 (15.1%)	0.579
Cerebrovascular diseases, (n, %)	3 (2.8%)	1 (1.9%)	2 (3.8%)	0.618
Obesity, (n, %)	13 (12.2%)	7 (13.0%)	6 (11.3%)	> 0.999
Hypertension, (n, %)	34 (31.8%)	21 (38.9%)	13 (24.5%)	0.146
Diabetes, (n, %)	10 (9.3%)	5 (9.3%)	5 (9.4%)	> 0.999
Apache II score	12 [11‒14]	12 [11‒14]	12 [11‒14]	0.801
Heart rate, (beats/min)	92 [81–107.5]	92 [82.5–106.5]	92. [80–107]	0.825
Mean Arterial Pressure, (mmHg)	82 [73–89]	82 [74–90.8]	83 [73–93]	0.886

**Fig. 2:**
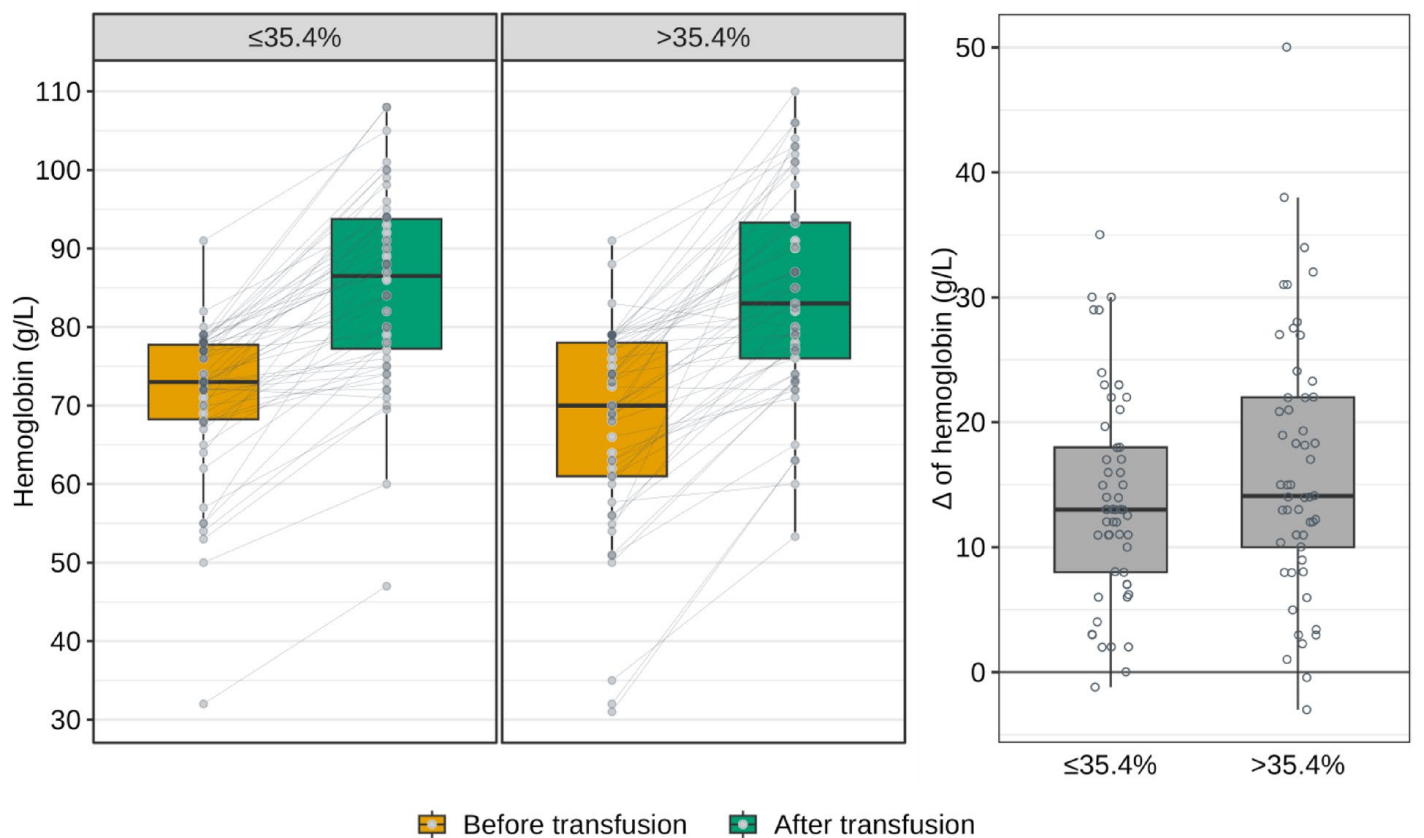
Hemoglobin levels (g/L) before and after transfusion in groups (left panels) and the magnitude of Δ hemoglobin (g/L) between the groups (right panel).

Hemoglobin levels increased significantly after transfusion in both groups (*p* < 0.001). Importantly, there were no statistically significant differences in baseline hemoglobin concentrations between the groups (*p* = 0.315), and the magnitude of post-transfusion change was also comparable (*p* = 0.214). Detailed distributions are presented in Fig. 2 and Supplementary Table S1. However, as by group definition, there were statistically significant differences in baseline levels of oxygen extraction ratio (O_2_ER), with concomitant differences in partial pressure of oxygen in central venous blood (PvO_2_), central venous oxygen saturation (ScvO_2_), arteriovenous oxygen difference (A-V O_2_diff), and veno-arterial carbon dioxide difference (ΔCO_2_) (Supplementary Table S1).

The dynamics of the studied parameters before and after blood transfusion in both groups are presented in Supplementary Table S1.

### Evaluation by groups

The median transfusion volume was similar in both groups: 335 mL [310–367.5] in the “Low O_2_ER” group and 340 mL [300–570] in the “High O_2_ER” group (*p* = 0.91). While most patients received one unit of red blood cells, 34 patients (31.8%) received two units. The decision to administer one or two units was based on baseline hemoglobin levels and followed the institutional transfusion protocol.

**Fig. 3:**
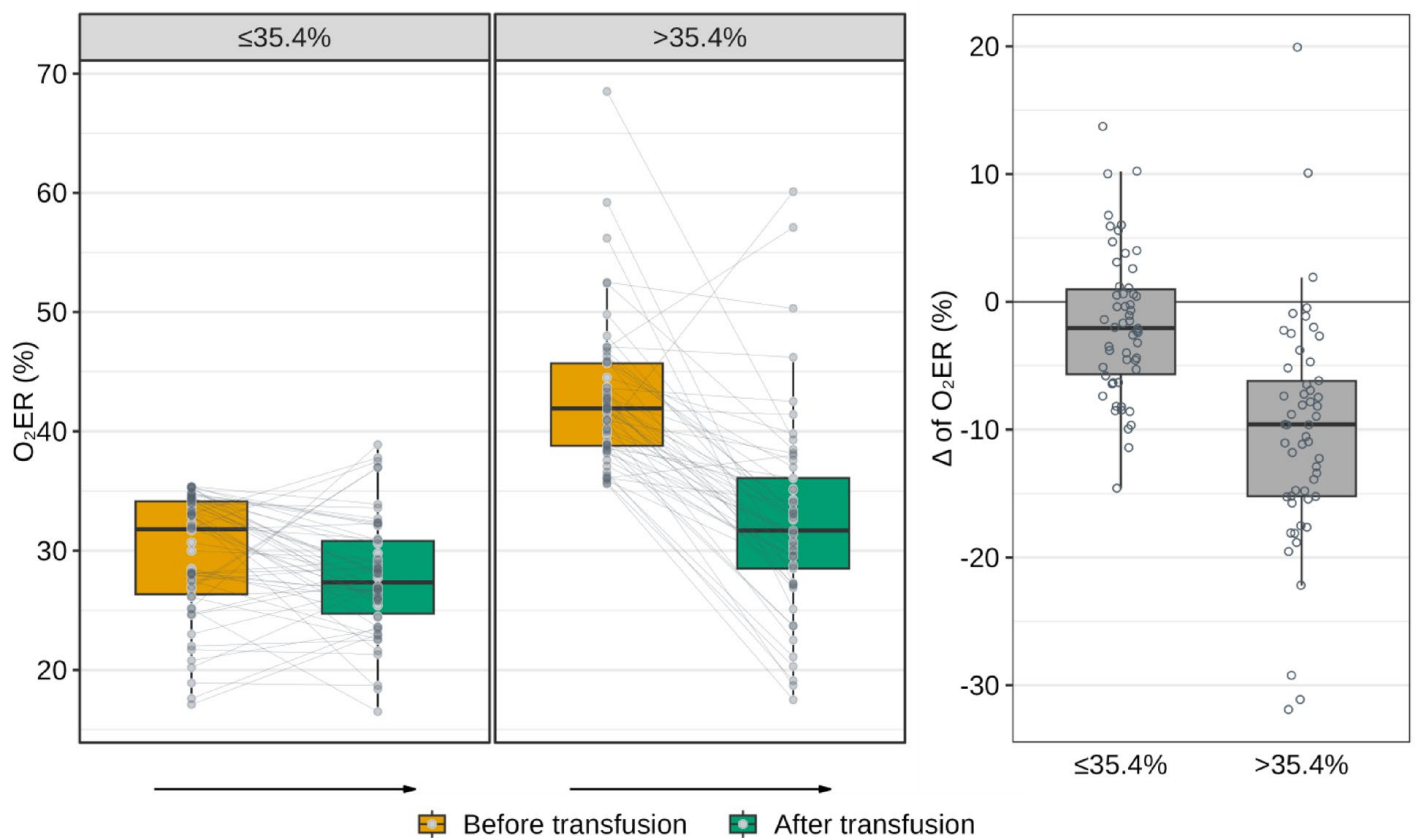
The dynamics of oxygen extraction ratio (O_2_ER) levels across groups before and after transfusion, and the dynamics of changes in O_2_ER after blood transfusion.

In both groups, O_2_ER significantly decreased after transfusion (*p* = 0.017 for the «Low O_2_ER», *p* < 0.001 for the «High O_2_ER»). However, the magnitude of this reduction was significantly greater in the group with higher baseline O_2_ER compared to the lower O_2_ER group (*p* < 0.001). Despite these improvements, O_2_ER values post-transfusion remained significantly higher in the second group compared to the first (*p* < 0.001) (Fig. 3).

Changes in physiological parameters are detailed in Supplementary Table S1. Regarding PvO_2_, the “High O_2_ER” group showed a significant increase following transfusion, whereas no significant change was observed in the “Low O_2_ER” group. Consequently, the improvement in PvO_2_ was significantly greater in the “High O_2_ER” group (*p* < 0.001). Similarly, ScvO_2_ increased significantly in both groups, but the magnitude of improvement was substantially greater in the “High O_2_ER” group compared to the “Low O_2_ER” group (*p* < 0.001). Lactate levels significantly decreased only in the “High O_2_ER” group, with no significant change in the “Low O_2_ER” group. The A-V O_2_ difference showed divergent responses: it increased in the “Low O_2_ER” group but significantly decreased toward normalization in the “High O_2_ER” group (p < 0.001 for the difference between groups). Finally, the ΔCO_2_ significantly decreased after transfusion in the “High O_2_ER, while remaining unchanged in the “Low O_2_ER” group (*p* = 0.001 for the difference).

The correlation analysis revealed significant associations between baseline O_2_ER and changes in PvO_2_ (*p* < 0.001), ScvO_2_ (*p* < 0.001), lactate levels (*p* = 0.028), A-V O_2_ difference (*p* < 0.001), and ΔCO_2_ (*p* = 0.001) after transfusion. In contrast, baseline hemoglobin concentrations were significantly correlated only with lactate changes (*p* < 0.001) and showed no significant association with other parameters. The correlation analysis results are presented in Supplementary Table S2.

## Discussion

Our study demonstrates that oncology patients with anemia and elevated baseline oxygen extraction ratios (O_2_ER > 35.4%) experienced significantly greater physiological improvements after blood transfusion compared to those with lower O_2_ER. Patients in the high O_2_ER group exhibited notable increases in central venous oxygen saturation (ScvO_2_), partial pressure of oxygen (PvO_2_), and reductions in lactate, arteriovenous oxygen difference, and veno-arterial CO_2_ gap. These results suggest that baseline O_2_ER effectively differentiates patients who physiologically benefit from transfusion, supporting its use as a clinical biomarker for targeted transfusion strategies aimed at optimizing oxygen delivery and avoiding unnecessary transfusions.

Our findings clearly demonstrate greater normalization of O_2_ER following transfusion in patients with elevated baseline levels, suggesting an improvement in the balance between oxygen delivery and consumption. Conversely, minimal improvements in the low-O_2_ER group suggest limited clinical relevance for transfusions in patients with adequate baseline tissue oxygenation. Lactate levels remained normal, likely because oxygen delivery was sufficient to maintain aerobic metabolism. As shown in previous studies, lactate accumulation typically begins only after oxygen extraction can no longer compensate for decreased DO_2_^27^.

We also observed statistically significant correlations between baseline levels of O_2_ER and ScvO_2_, PvO_2_, A-V O_2_ difference, lactate, ΔCO_2_, and their changes following transfusion. These associations highlight the physiological impact of transfusion, reinforcing the importance of using physiologically driven parameters rather than isolated hemoglobin thresholds.

Our findings support a shift toward personalized transfusion strategies that move beyond the “one-size-fits-all” hemoglobin threshold paradigm and instead incorporate dynamic physiological parameters to guide decision-making^26,28,29^. Incorporating such parameters into clinical protocols could enhance patient safety by minimizing unnecessary transfusions and reducing transfusion-associated complications. Despite similar hemoglobin improvements post-transfusion, differential physiological responses between groups further support the importance of O_2_ER in assessing transfusion necessity.

Our findings are in line with those reported by previous studies. Vallet et al. demonstrated the effectiveness of ScvO_2_ as a transfusion trigger^30,31^, while Surve et al. demonstrated that baseline central venous oxygen saturation (ScvO_2_) levels effectively predicted significant physiological improvements following blood transfusion in neurointensive care patients, highlighting ScvO_2_ as a valuable physiological transfusion trigger compared to hemoglobin-based approaches^32^. We found no correlation between ScvO_2_ and baseline hemoglobin levels, possibly due to hemoglobin’s limited reflection of true oxygen delivery^26^. While O_2_ER provides a precise physiological index of the balance between oxygen delivery and consumption, we acknowledge that its calculation requires paired arterial and central venous blood gas analyses. Given the strong physiological coupling between O_2_ER and ScvO_2_, the latter can serve as a practical surrogate for guiding transfusion decisions in hemodynamically stable patients. However, it is important to recognize that ScvO_2_ is determined not only by hemoglobin concentration but also by arterial oxygen saturation, cardiac output, and oxygen consumption^33^. Therefore, it should be used with caution in patients with arterial hypoxemia or low cardiac output, in whom O_2_ER provides a more comprehensive assessment of tissue oxygenation.

Additionally, Fogagnolo et al. showed improved survival when transfusion was guided by elevated arteriovenous oxygen differences^34^. Orlov et al. demonstrated that O_2_ER-guided strategies reduced unnecessary transfusions in cardiac surgery without compromising safety^35^.

The selected cut-off value for O_2_ER (> 35.4%) was based on the median value observed in our study population and was intentionally set above normal physiological levels (approximately 25–30%) to better capture impaired oxygen extraction. Our threshold falls within the range reported in previous studies. For instance, Fogagnolo et al. used a similar median-split approach^34^. Tüzen et al. utilized a threshold of 30%, whereas Ranucci et al. identified a critical threshold of 39% in cardiac surgery patients^36,37^. As highlighted in a recent review by Hess, establishing a universal physiological trigger remains challenging due to population variability, reinforcing the value of physiological markers over static hemoglobin thresholds^38^. In our cohort, patients with baseline O_2_ER values above the median (35.4%) demonstrated more pronounced physiological improvement following transfusion. While this threshold was not pre-specified or formally validated, it may serve as a reference point for further studies exploring O_2_ER-guided transfusion strategies.

Our study confirms that incorporating O_2_ER into transfusion decisions enables more accurate identification of patients with true oxygen deficits. This physiology-based approach supports a more rational allocation of red blood cells, aligning with contemporary patient blood management principles and promoting both safety and efficiency.

Several limitations should be noted. First, the observational nature of our study introduces potential selection bias and unmeasured confounding factors, including those that may influence oxygen extraction (e.g., sepsis). However, it should be noted that both groups demonstrated comparable hemodynamic stability at baseline. Second, the study included only stable patients, limiting generalizability. Third, the single-center design may restrict applicability to other settings. Finally, this study was not designed or powered to detect differences in mortality or other adverse clinical outcomes. Further research involving diverse patient populations is required to validate these findings.

Despite these limitations, the study’s strengths include a comprehensive evaluation of multiple physiological transfusion triggers and the stratification based on O_2_ER, enabling precise identification of patients likely to benefit from transfusions. By analyzing dynamic oxygenation parameters rather than relying solely on hemoglobin thresholds, the study highlights the clinical relevance of functional indicators in transfusion decision-making. These strengths underscore the growing importance of personalized, physiology-driven transfusion strategies in optimizing patient outcomes and minimizing unnecessary interventions. Moreover, given the growing emphasis on individualized patient care, integrating physiological transfusion triggers into routine clinical practice may represent a critical step toward safer and more effective blood management strategies.

## Conclusion

Despite the widespread reliance on hemoglobin levels as the primary transfusion trigger among clinicians, it may not accurately reflect the adequacy of oxygen delivery in individual patients. In a cohort of oncological patients, we showed that baseline oxygen extraction ratio (O_2_ER) was associated with the magnitude of physiological improvement after transfusion, including changes in ScvO_2_, PvO_2_, lactate, and other oxygenation indices. This suggests that O_2_ER may help identify patients with true oxygen deficits who are more likely to benefit from transfusion. Parameters reflecting oxygen delivery may be valuable in guiding decisions regarding blood transfusion. Further large-scale clinical studies are needed to evaluate the safety and clinical outcomes of using alternative transfusion triggers.

## Supplementary Information

Below is the link to the electronic supplementary material.

Supplementary Material 1

## Data Availability

The datasets supporting the conclusions of this article are available from the corresponding author on request, without restriction.

## Abbreviations

A-V O_2_ diff: Arteriovenous oxygen difference
BMI: Body mass index
CtO_2_: Oxygen content
CtaO_2_: Arterial oxygen content
CtvO_2_: Central venous oxygen content
ΔCO_2_: Veno-arterial carbon dioxide difference
ESAIC: European society of Anaesthesiology and Intensive Care
Hb: Hemoglobin
O_2_ER: Oxygen extraction ratio
PaCO_2_: Partial pressure of carbon dioxide in arterial blood
PaO_2_: Partial pressure of oxygen in arterial blood
PvCO_2_: Partial pressure of carbon dioxide in central venous blood
PvO_2_: Partial pressure of oxygen in central venous blood
ScvO_2_: Central venous oxygen saturation
