# Supplementary material for: Evaluation of alternative transfusion triggers in hemodynamically stable, non-ventilated cancer patients: a prospective observational study
Source: Sci Rep. 2025 Dec 19;16:2831. doi: 10.1038/s41598-025-32630-6 (PMC12824186; doi:10.1038/s41598-025-32630-6)
Supplement: Supplementary file 1 — Supplementary Material 1 [file 41598_2025_32630_MOESM1_ESM.docx]

**Supplementary Materials**

**Supplementary Table S1. Dynamics of Parameters Before and After Transfusion in both groups**

| Parameters | "Low O_2_ER" (≤35.4%), n = 54 | "High O_2_ER" (>35.4%), n = 53 | p-value |
| --- | --- | --- | --- |
| O_2_ER, (%) |  | | |
| Before transfusion | 31.8 [26.4‒34.2] | 41.9 [38.8‒45.7] | - |
| After transfusion | 27.4 [24.7‒30.8] | 31.7 [28.5‒36.1] | <0.001 |
| Δ | -2.1 [-5.7‒1] | -9.6 [-15.2‒ -6.2] | <0.001 |
| Hemoglobin, (g/l) |  | | |
| Before transfusion | 73 [68.3‒77.8] | 70 [61‒78] | 0.315 |
| After transfusion | 86.5 [77.3‒93.8] | 83 [76‒93.3] | 0.683 |
| Δ | 13 [8‒18] | 14.1 [10‒22] | 0.214 |
| PvO_2_ (mmHg) |  | | |
| Before transfusion | 37.8 [35.5‒39.7] | 33.3 [30.8‒34.3] | <0.001 |
| After transfusion | 38 [35.5‒39.3] | 34.3 [32.6‒37.2] | <0.001 |
| Δ | 0.05 [-2.8‒2.18] | 2.1 [0.5‒4.3] | <0.001 |
| ScvO_2_ (%) |  | | |
| Before transfusion | 68.1 [63.2‒71.5] | 55.9 [54.3‒60.8] | <0.001 |
| After transfusion | 70.2 [67.5‒74] | 65.7 [61.8‒69.7] | <0.001 |
| Δ | 1.9 [-1‒6] | 9 [5.4‒13.8] | <0.001 |
| Lactate, (mmol/l) |  | | |
| Before transfusion | 1.2 [0.9‒1.7] | 1.1 [0.9‒1.7] | 0.657 |
| After transfusion | 1.1 [0.7‒1.8] | 0.9 [0.6‒1.3] | 0.136 |
| Δ | -0.05 [-0.28‒0.3] | -0.2 [-0.6‒0] | 0.006 |
| A-V O_2_diff, (%) |  | | |
| Before transfusion | 28 [24.7‒32.2] | 39.3 [34.2‒44] | <0.001 |
| After transfusion | 31.4 [26.4‒35.6] | 33.6 [27.9‒40.2] | 0.043 |
| Δ | 3.2 [-1.7‒7.5] | -3.2 [-9.7‒1.9] | <0.001 |
| ΔCO_2_ (mmol/l) |  | | |
| Before transfusion | 6.1 [4.5‒7.8] | 7.6 [6.5‒9.6] | <0.001 |
| After transfusion | 6.6 [4.8‒7.8] | 6 [5‒7.5] | 0.77 |
| Δ | -0.3 [-1.7‒2.3] | -1.5 [-3.3‒0] | 0.001 |

**Supplementary Table S2. Correlation between baseline parameters and changes after transfusion.**

| Section | Parameter change after transfusion | ρ (Spearman) | 95 % CI | p‑value |
| --- | --- | --- | --- | --- |
| A. Baseline O₂ER | Δ PvO₂ | 0.39 | 0.22; 0.54 | < 0.001 |
|  | Δ ScvO₂ | 0.59 | 0.45; 0.70 | < 0.001 |
|  | Δ Lactate | –0.21 | –0.39; -0.02 | 0.028 |
|  | Δ A‑V O₂ diff | –0.46 | –0.60; 0.30 | < 0.001 |
|  | Δ ΔCO₂ | –0.31 | –0.47; 0.12 | 0.001 |
| B. Baseline Hemoglobin | Δ PvO₂ | -0.10 | -0.28; 0.10 | 0.326 |
|  | Δ ScvO₂ | -0.13 | -0.32; 0.06 | 0.171 |
|  | Δ Lactate | 0.35 | 0.17; 0.51 | <0.001 |
|  | Δ A‑V O₂ diff | -0.07 | -0.26; 0.12 | 0.457 |
|  | Δ ΔCO₂ | -0.31 | -0.07; 0.31 | 0.199 |
